# Activation of CB1R alleviates central sensitization by regulating HCN2-pNR2B signaling in a chronic migraine rat model

**DOI:** 10.1186/s10194-023-01580-7

**Published:** 2023-04-21

**Authors:** Xiaoxu Zeng, Jia Mai, Hongjian Xie, Ling Yang, Xiaojuan Liu

**Affiliations:** 1 Department of Laboratory Medicine, West China Second University Hospital, Sichuan University, Chengdu, China; 2 Key Laboratory of Birth Defects and Related Diseases of Women and Children, Sichuan University, Ministry of Education, Chengdu, China

**Keywords:** Chronic migraine, Cannabinoid receptor, HCN, NR2B, Central sensitization

## Abstract

**Background:**

Central sensitization has been widely accepted as an underlying pathophysiological mechanism of chronic migraine (CM), activation of cannabinoid type-1 receptor (CB1R) exerts antinociceptive effects by relieving central sensitization in many pain models. However, the role of CB1R in the central sensitization of CM is still unclear.

**Methods:**

A CM model was established by infusing inflammatory soup (IS) into the dura of male Wistar rats for 7 days, and hyperalgesia was assessed by the mechanical and thermal thresholds. In the periaqueductal gray (PAG), the mRNA and protein levels of CB1R and hyperpolarization-activated cyclic nucleotide-gated cation channel 2 (HCN2) were measured by qRT–PCR and western blotting. After intraventricular injection of Noladin ether (NE) (a CB1R agonist), ZD 7288 (an HCN2 blocker), and AM 251 (a CB1R antagonist), the expression of tyrosine phosphorylation of N-methyl-D-aspartate receptor subtype 2B (pNR2B), calcium-calmodulin-dependent kinase II (CaMKII), and phosphorylated cAMP-responsive element binding protein (pCREB) was detected, and central sensitization was evaluated by the expression of calcitonin gene-related peptide (CGRP), c-Fos, and substance P (SP). Synaptic-associated protein (postsynaptic density protein 95 (PSD95) and synaptophysin (Syp)) and synaptic ultrastructure were detected to explore synaptic plasticity in central sensitization.

**Results:**

We observed that the mRNA and protein levels of CB1R and HCN2 were both significantly increased in the PAG of CM rats. The application of NE or ZD 7288 ameliorated IS-induced hyperalgesia; repressed the pNR2B/CaMKII/pCREB pathway; reduced CGRP, c-Fos, SP, PSD95, and Syp expression; and inhibited synaptic transmission. Strikingly, the application of ZD 7288 relieved AM 251-evoked elevation of pNR2B, CGRP, and c-Fos expression.

**Conclusions:**

These data reveal that activation of CB1R alleviates central sensitization by regulating HCN2-pNR2B signaling in CM rats. The activation of CB1R might have a positive influence on the prevention of CM by mitigating central sensitization.

## Background

Chronic migraine (CM) is defined as a headache that occurs at least 15 days a month for more than three months [1], and usually develops from episodic migraine. Chronic migraine has a very adverse impact on the life of patients, can seriously damage their social and economic function and quality of life and is a highly disabling disease [2, 3]. However, the pathogenesis of CM is still poorly understood. Emerging evidence indicates that central sensitization is the underlying pathophysiological mechanism of CM [4]. Central sensitization represents an increase in the excitability of neurons and the strengthened synaptic efficacy in the nociceptive pathway in response to noxious stimuli [5, 6].

Synaptic plasticity has been widely accepted as a vital mechanism for the development of central sensitization [7, 8]. Synaptic plasticity refers to changes in the morphology and function of synapses, which result in the enhancement of synaptic transmission efficiency [9]. N-methyl-D-aspartate receptor subtype 2B (NR2B) is a subunit of the ionotropic glutamate N-methyl-D-aspartate (NMDA) receptor, tyrosine phosphorylation of NR2B (pNR2B) is the activated form of the NMDA receptor, which plays a decisive role in controlling the influx of Ca^2+^ through the NMDA receptor, regulating synaptic transmission, and determining the properties of the NMDA receptor [10, 11]. Spinal administration of a selective NR2B-containing NMDA receptor antagonist relieves neuropathic pain without leading to motor dysfunction and inhibits the induction of long-term potentiation (LTP), a form of synaptic plasticity, and nociceptive stimulation-triggered activity in the spinal dorsal horn, suggesting the activation of NR2B in the spinal cord may participate in central sensitization and neuropathic pain by inducing LTP of nociceptive synaptic transmission in the spinal dorsal horn [12]. In our previous research, the phosphorylation of NR2B facilitated central sensitization by modulating synaptic plasticity in the trigeminal nucleus caudalis of CM rats [13].

Hyperpolarization-activated cyclic nucleotide–gated (HCN) channels, the tetrameric voltage-controlled ion channels in the cell membrane of neurons, contribute to nociceptive excitability [14]. The HCN family consists of four HCN subtypes (HCN1 to HCN4), which are expressed in sensory neurons, and over half of the small nociceptive neurons express HCN2 channels [15, 16]. Recently, HCN2 has been widely studied because of its new status as an analgesic target in pathological pain. When HCN2 was inhibited in primary sensory neurons, hypersensitivity was significantly relieved in chronic inflammation [17]. In addition, studies have reported that HCN2 contributes to the induction of spinal LTP at C-fiber synapses in oxaliplatin-related neuropathic pain by regulating NR2B, and this process is achieved by activating the calcium-calmodulin-dependent kinase II (CaMKII)/cAMP-responsive element binding protein (CREB) cascade [18, 19], reflecting that HCN2 and NR2B may be closely related in the conduction and regulation of pain.

The endocannabinoid system, consisting of the endogenous cannabinoid ligands (endocannabinoids), cannabinoid type-1 receptor (CB1R) and cannabinoid type-1 receptor (CB2R), and metabolizing enzymes, is present throughout the pain pathways [20]. CB1R is a Gi/o protein-coupled receptor, and the CB1R agonists have antinociceptive effects in animal models of neuropathic, inflammatory, and acute pain [21, 22]. Strikingly, a recent study reported that in the hippocampus, CB1R regulates synaptic plasticity and spatial memory formation via HCN channels that underlie the h-current (hI), a key regulator of dendritic excitability [23]. When the CB1R-HCN pathway is activated, dendritic integration of excitatory inputs, LTP, and spatial memory formation are all weakened [24]. However, the roles and relationships of CB1R and HCN in CM have not been explored thus far.

In animal models, stimulation of dural vessels leads to the activation of neurons in the spinal trigeminal nucleus caudalis (TNC) and the C1 and C2 regions of the cervical spinal cord, collectively referred to as the trigeminocervical complex (TCC) [25]. The severe pain of migraine is due to the activation of these nociceptive inputs from both intracranial and extracranial structures, which converge and are transmitted through the TCC [26]. The periaqueductal gray (PAG) is the origin of the “descending inhibitory system” [27, 28]. There is direct descending modulation of TCC neurons from projections from the PAG that pass through the rostral ventromedial medulla (RVM) [25, 29]. Furthermore, functional studies have shown that microinjection of cannabinoids into the PAG produces antinociceptive nociception [30, 31]. Therefore, we propose a hypothesis that the activation of CB1R relieves central sensitization by modulating HCN2-pNR2B signaling in the PAG of CM rats.

## Materials and methods

### Animals

**Fig. 1:**
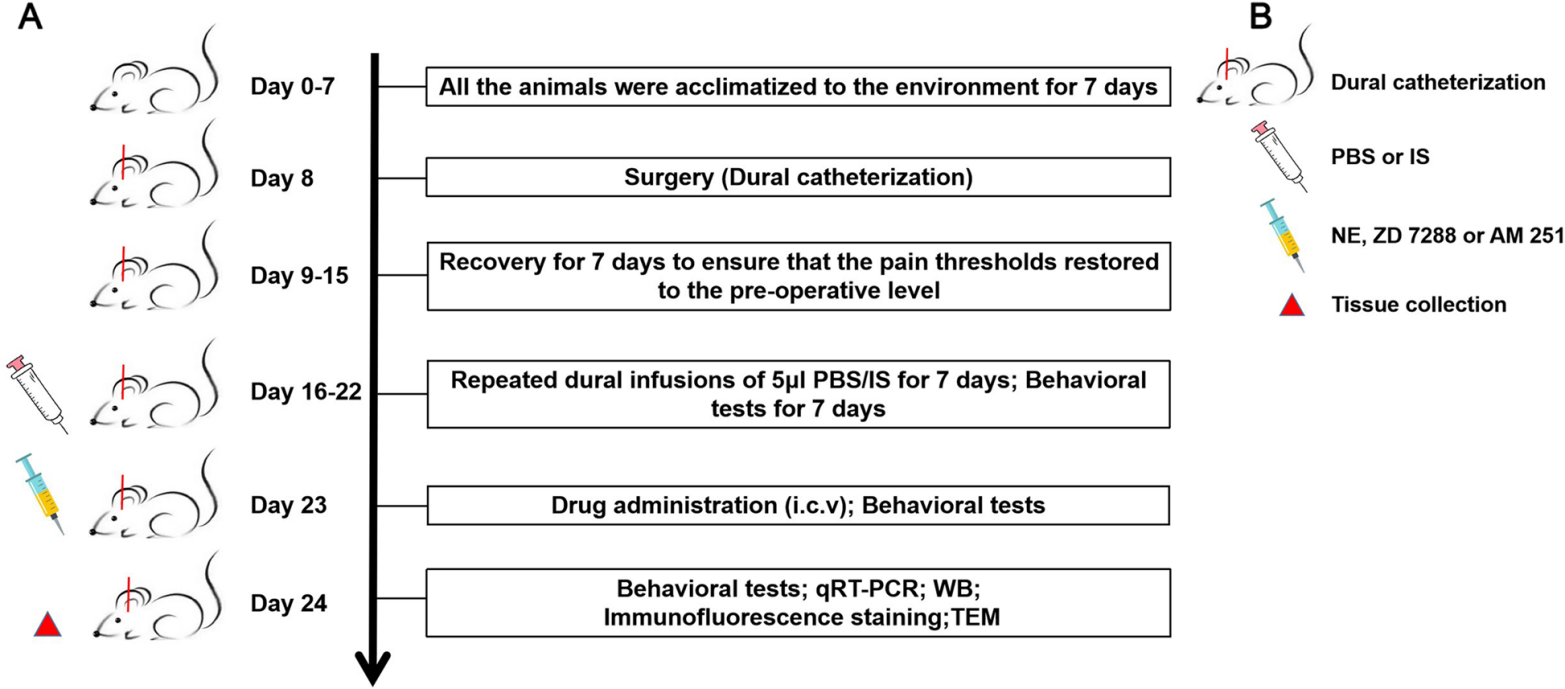
The timeline and experimental steps in this research. Rats were randomly assigned to the experimental groups after 7 days of acclimatization. On the 8th day, rats underwent surgery for the establishment of the CM model, and rats with dura damage during the surgery were excluded. From the 9th day, rats were allowed to recover for 7 days to ensure that the pain thresholds were restored to the preoperative level. From the 16th day, rats were subjected to dural infusions of 5 μl of PBS/IS and behavioral tests for 7 days. On the 23rd day, rats were treated with intraventricular drug injections, and behavioral tests were performed. On the 24th day, after behavioral tests were performed, and rats were euthanized for the qRT–PCR, western blotting, immunofluorescence staining, and TEM

A total of 208 healthy adult male Wistar rats (250-300 g; specific pathogen-free; certificate no. SCXK [LIAO] 2015–0001; Liaoning, China) were used to conduct experiments because the frequency of migraine attacks is affected by estrogen level [32]. Rats were obtained from Liaoning Changsheng Biotechnology Co., Lt (Benxi, Liaoning, China). Rats were housed in a temperature-controlled (23 ± 1 °C) room with a 12-h light/dark cycle and enough water and food. All the procedures conformed to the National Institutes of Health Guide for the Care and Use of Laboratory Animals. All experiments conducted on the rats were approved by the Ethics Committee of the Department of Medical Research (First Affiliated Hospital of Chongqing Medical University). The timeline and experimental steps of this research are shown in Fig. 1.

### CM model (surgery and IS infusion)

In reference to previous procedures [33, 34], after rats were anesthetized with 10% chloral hydrate (4 ml/kg, intraperitoneal), buprenorphine was injected for analgesia (0.01 mg/kg, subcutaneously). A stereotaxic frame (ST-51603; Stoelting Co, Chicago, IL, USA) was used to fix the rat’s head. Afterward, an incision (approximately 2 cm long, along the line from the midpoint of the eyes to the midpoint of the ears) was made to fully expose the skull following disinfection with iodine. A burr drill was used to carefully perform craniotomy with a diameter of 1 mm above the left dura (-1.0 mm rear from bregma, + 1.5 mm lateral to bregma). Next, a stainless-steel cannula with a plastic cap was fixed on the skull with dental cement. After the incision was sutured, a temperature-controlled electric blanket was used to help the rats to wake up. To prevent the shedding of cannula caused by fighting between rats, the rats were fed singly for a week during which the wound was disinfected daily with iodophor. After ensuring that the pain thresholds of the rats returned to the basal levels, rats underwent dural infusion. By using the cannula that connects the dura, rats in the Sham group were infused with 5 μl of phosphate-buffered saline (PBS) (0.1 M, pH 7.4) for 7 consecutive days, and rats in the CM group were infused with 5 μl of inflammatory soup (IS) for 7 consecutive days. The IS was composed of 1 mM bradykinin, 1 mM serotonin, 1 mM histamine, and 0.1 mM prostaglandin E2, which were dissolved in PBS. All the above products were provided by Sigma (Missouri, USA).

### Drug administration

To reveal the specific participation mechanism of CB1R and HCN2 in CM, a CB1R agonist Noladin ether (NE) (MedChemExpress, New Jersey, USA), a CB1R antagonist AM 251 (MedChemExpress, New Jersey, USA), or an HCN blocker ZD 7288 (MedChemExpress, New Jersey, USA) was dissolved in vehicle (dimethyl sulphoxide (DMSO)-saline solution mixture (5:95)), and the customized dose of NE (3.6 μg and 36 μg), AM 251 (4 μg and 40 μg), or ZD 7288 (0.5 μg and 5 μg) was administered intraventricularly as described previously [18, 35, 36] after 7th dural infusion of PBS or IS (-1.0 mm rear from the bregma, + 1.5 mm lateral to the bregma, 4.0 mm from the skull plane; The microsyringe was used to pierce the dura mater through the cannula and reach the lateral ventricle). Each drug was injected intraventricularly in a volume of 10 μl (1ul/min) [37, 38]. As a control, an equal volume of vehicle was injected into the lateral ventricle. On the basis of the experimental design, rats were randomly divided into fourteen groups and the number of rats used in each group is shown in Table 1. Importantly, in the CM + AM 251 + ZD 7288 group, to prevent direct interaction of the two drugs, ZD 7288 was conducted at 30 min after AM 251 injection.

**Table 1:**
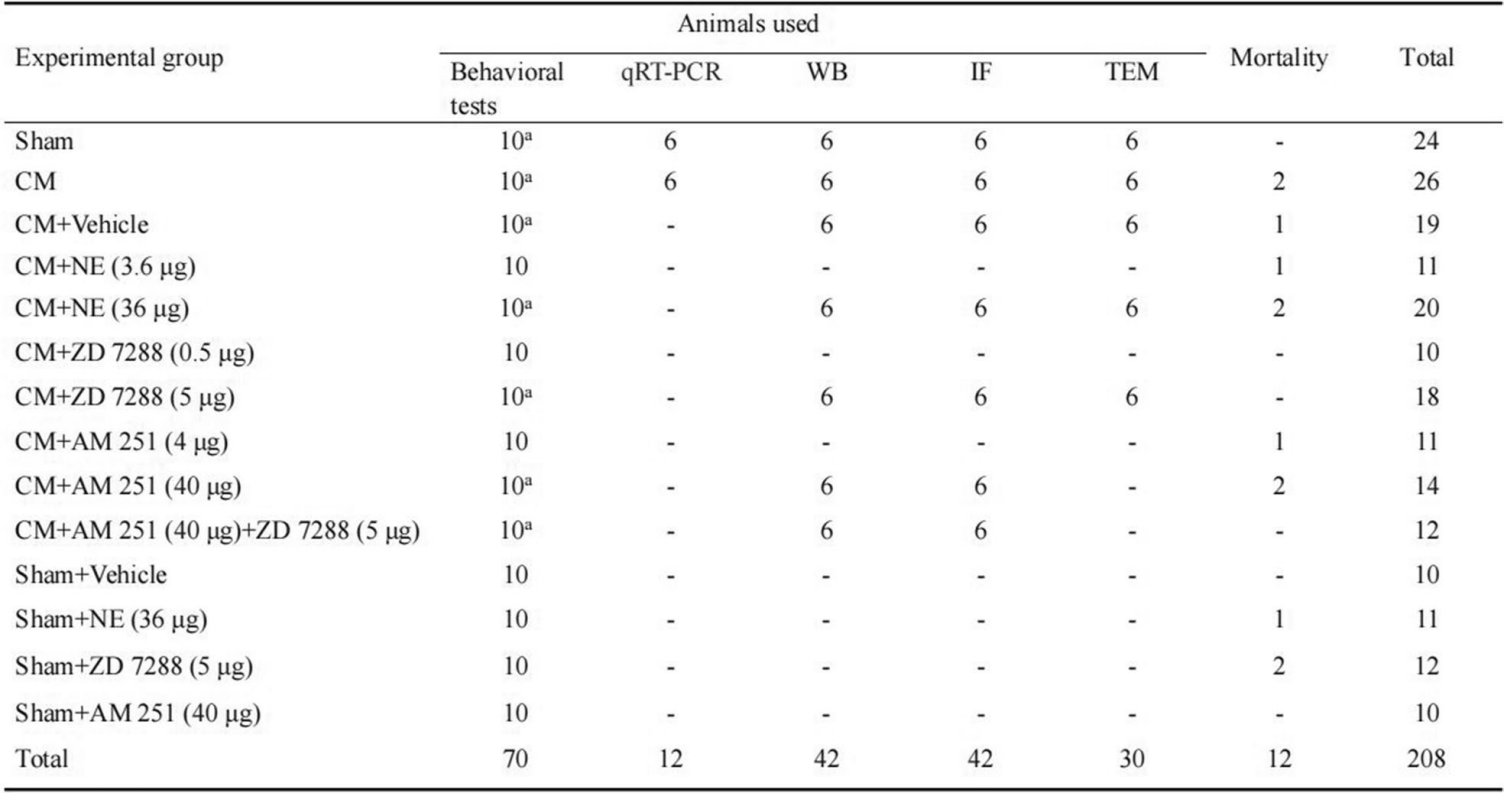
Animal numbers in each group

^a^Indicates shared with other experiments, do not count

### Behavioral tests

Mechanical and thermal pain threshold detection was performed before the first PBS/IS infusion, at 24 h after each PBS/IS infusion, and at 24 h after intraventricular drug injection. All animal behavior tests were performed under light conditions between 09:00 and 15:00 by an experimenter blinded to the experimental groups.

Mechanical thresholds of the hindpaw or periorbital region were detected using an electronic von Frey monofilament (Electrovonfrey, 2391, IITC Inc., Woodland Hills, CA, USA) [39]. The rats were acclimated in a transparent testing box for 30 min. Then the pressure probe tip was employed with increasing force to the middle of the plantar surface of the left hindpaw [40] or the right side of the face over the rostral portion of the eye of each rat [41]. A positive response was recorded when the rat’s left hindpaw or head quickly moved or retracted away from the probe tip. The mechanical pain thresholds were recorded automatically, and the test was repeated at least three times in each rat with an interval of at least 5 min. The average of the three results was calculated, which was identified as the mechanical pain threshold.

Referring to the method described previously [42], the thermal thresholds of the hindpaw were measured by the plantar test apparatus (Hargreaves test) (TechmanPL-200, Chengdu, China). The animals were acclimated in a transparent testing box for at least 30 min. Then, radiant heat was used at the middle of the plantar surface of the right hindpaw. A positive response was judged when the rats lifted their hindpaws away from the radiant heat or licked their hindpaws. The thermal pain thresholds of the hindpaw were judged automatically, and the test was repeated at least three times in each rat with an interval of at least 5 min. The average of the three results was calculated, which was identified as the thermal pain threshold. An automatic 30-s cutoff time was set to prevent the rats from getting burned.

### Quantitative real–time polymerase chain reaction (qRT–PCR)

With reference to the stereoscopic map of the rat brain, after the rats were euthanized, the rat brain was separated from the skull and placed on ice. Then, the cerebellum was removed with a blade to expose the midbrain aqueduct. Finally, the gray area around the midbrain aqueduct, the PAG, was isolated with a blade and tweezers. The PAG was stored in liquid nitrogen for subsequent analysis. Total RNA was extracted from the frozen PAG by RNAiso Plus reagent (TaKaRa, Dalian, China), and reverse transcription was performed using the PrimeScriptTM RT Reagent Kit (TaKaRa, Dalian, China). Next, The quantification of CB1R and HCN2 gene expression was determined on a CFX96 Touch thermocycler (Bio-Rad, USA) using SYBR Premix Ex TaqTM II (TaKaRa, Dalian, China). The CB1R, CB2R, HCN1, HCN2, HCN3, and HCN4 gene expression was normalized to the GAPDH gene. Specific primers of CB1R, CB2R, HCN1, HCN2, HCN3, HCN4, and GAPDH obtained from Sangon Biotech (Shanghai, China) were described as follows: CB1R: 5'-TGG CTC TGT TTA GAT GTT TGG-3' (forward), 5'-GAG AAC CTG TAT GAG GAG AG-3' (reverse); CB2R: 5' -GCC TGG TCA TGG CTG TTC TG-3' (forward), 5'-CAG CAG AGC GGA TCT CTC CA-3' (reverse); HCN1: 5'-CTC TTA CTT TGG AGA AAT ATG C-3' (forward), 5'-TAG AGT TTT TCT TGC CTA TCC G-3' (reverse); HCN2: 5'-GGG AAT CGA CTC CGA GGT CTA C-3' (forward), 5'-AGA CTG AGG ATC TTG GTG AAA CG-3' (reverse); HCN3: 5'-GAT ACT GCA GCG GAA ACG CTC-3' (forward), 5'-AGA TAC CTG GGA ACG CCC TGT-3' (reverse); HCN4: 5'-CCC GCC TCA TTC GAT ACA TTC AT-3' (forward), 5'-GAG GGC GTA GGA ATA CTG CTT C-3' (reverse); GAPDH: 5'-ATG ACT CTA CCC ACG GCA AGC T-3' (forward), 5'-GGA TGC AGG GAT GAT GTT CT-3' (reverse). The standard Δ Δ Cq method was applied to analyze gene expression.

### Western blotting (WB)

After the rats were sacrificed, the PAG tissue was removed. The PAG was homogenized in a radioimmunoprecipitation assay (RIPA) lysis buffer (Beyotime, Shanghai, China) with protease inhibitor PMSF (Beyotime, Shanghai, China) at 4 °C for 1 h. The Bicinchoninic Acid (BCA) protein analysis kit was employed to analyze protein concentration (Beyotime, Shanghai, China). The protein samples were electrophoresed on an SDS-PAGE gel (Beyotime, Shanghai, China), and transferred to a polyvinylidene difluoride (PVDF) membrane (Millipore, USA). Then, the membranes were blocked with 5% non-fat milk for 2 h at room temperature, in turn, incubated overnight at 4 °C with the primary antibodies diluted in TBST. The next day, the secondary antibodies were used at 37 °C for 1 h. Then, the membranes were visualized in an imaging system (Fusion, Germany) with the Beyo ECL Plus kit (Beyotime, Shanghai, China). The β-actin or GAPDH was used as a control to normalize protein expression. Detailed antibody information used in western blotting is shown in Table 2.

**Table 2:**
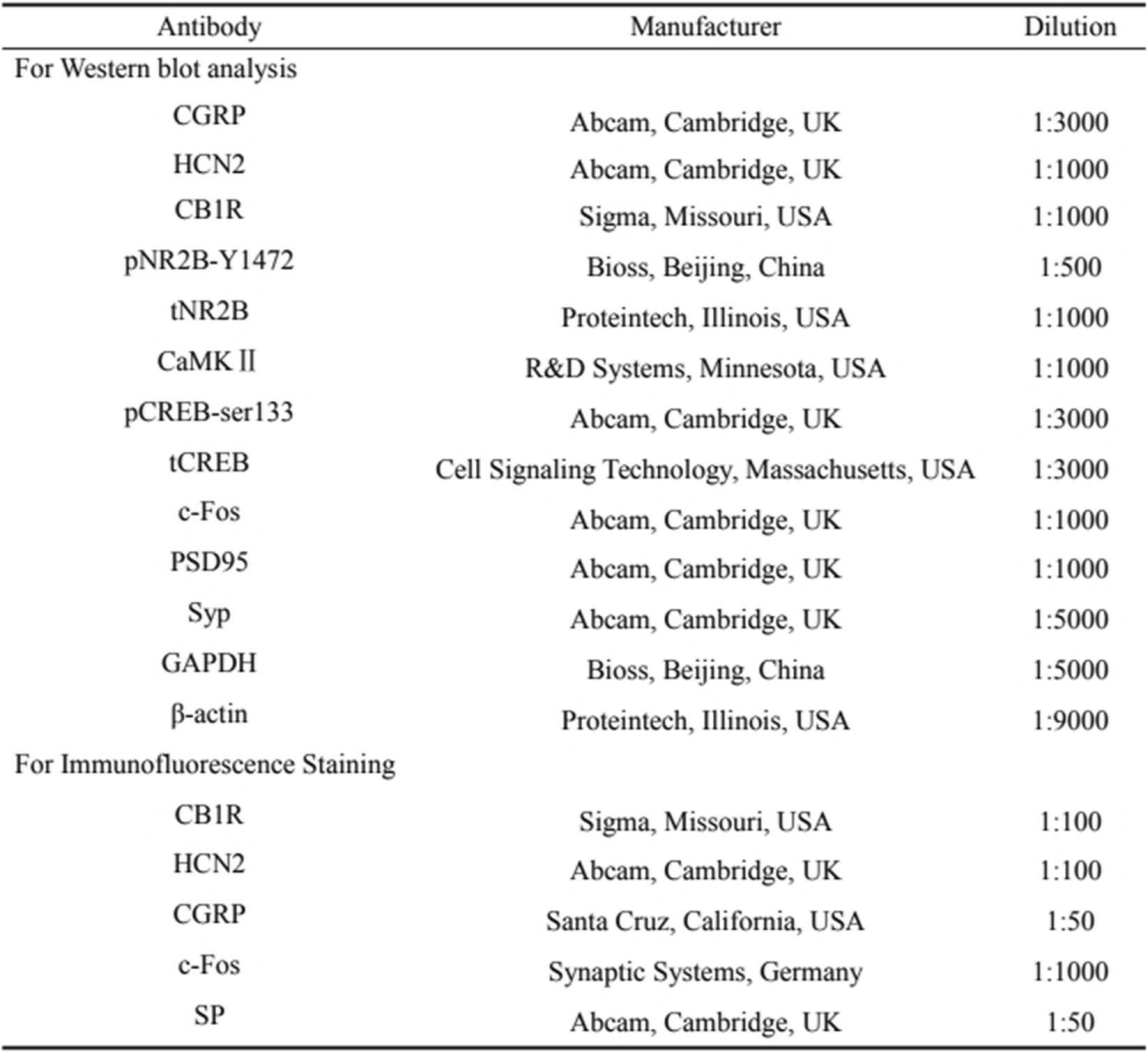
Antibodies used in Western blotting and immunofluorescence staining

### Immunofluorescence staining

After anesthetized, rats were perfused transcardially with 4% paraformaldehyde. The PAG tissue was isolated immediately following perfusion, then post-fixed in 4% paraformaldehyde at 4 °C for 24 h, and subsequently transferred to sucrose with increasing concentrations (20–30%) until it sank. Afterward, the PAG was sliced transversely at 10 μm using a freezing microtome (Leica, Japan), and the tissue sections were collected and secured on glass slides. After undergoing antigen retrieval with sodium citrate (Beyotime, Shanghai, China), the sections were incubated with 10% goat serum (Boster, Wuhan, China) at 37 °C for 30 min, then incubated overnight at 4 °C with the primary antibodies diluted in PBS. The next day, the secondary antibody was employed at 37 °C for 1.5 h. Whereafter, the sections were incubated with 4′,6-diamidino-2-phenylindole (DAPI) (Beyotime, China) at 37 °C for 10 min. The images were captured under the confocal laser scanning fluorescence microscope (ZEISS, Germany). Image-J was used to analyze the fluorescence intensity. The analysts were blinded to the experimental groups. Detailed antibody information used in immunofluorescence staining is shown in Table 2.

### Transmission electron microscopy (TEM)

Rats were perfused transcardially with 2.5% glutaraldehyde following anesthetization. The PAG and its surrounding extensions were separated immediately after perfusion, then post-fixed in 4% glutaraldehyde at 4 °C for 24 h. Next, a sharp blade was used to cut the PAG tissues into 1 mm pieces, which were sent to Chongqing Medical University for subsequent fixation, embedding, slicing, and staining according to the method as described previously [43]. The images were taken using an EM-1400 PLUS transmission electron microscope (TEM) and statistical analysis was implemented with Image-pro Plus 6.2. The thickness of the postsynaptic density, the length of the synaptic activity zone, the width of the synaptic cleft, and the curvature of the synaptic interface, four related indicators of the synaptic ultrastructure, were measured precisely. The length of the synaptic activity zone and the thickness of the PSD were detected based on the previous methods [44], the multipoint averaging method was performed to measure the width of the synaptic cleft and the curvature of the synaptic interface was measured according to the description of Jones and Devon [45]. The evaluations were performed by an observer blinded to the experimental groups.

### Statistical analysis

All the data represent the mean ± SD in this article. All the data were tested for normality by the Kolmogorov–Smirnov (K-S) normality tests. Graphs were generated by GraphPad Prism 7. SPSS 20.0 was employed for statistical evaluations. Statistical differences in pain thresholds were evaluated using a two-way analysis of variance followed by a Bonferroni post hoc test. Statistical differences between the two groups were assessed using independent-sample t-tests. A one-way analysis of variance followed by the Bonferroni post hoc test was used to analyze multiple comparisons. A significance level of *p* < 0.05 was used.

## Results

### The influence of repeated dural IS stimulation on hyperalgesia in CM rats

**Fig. 2:**
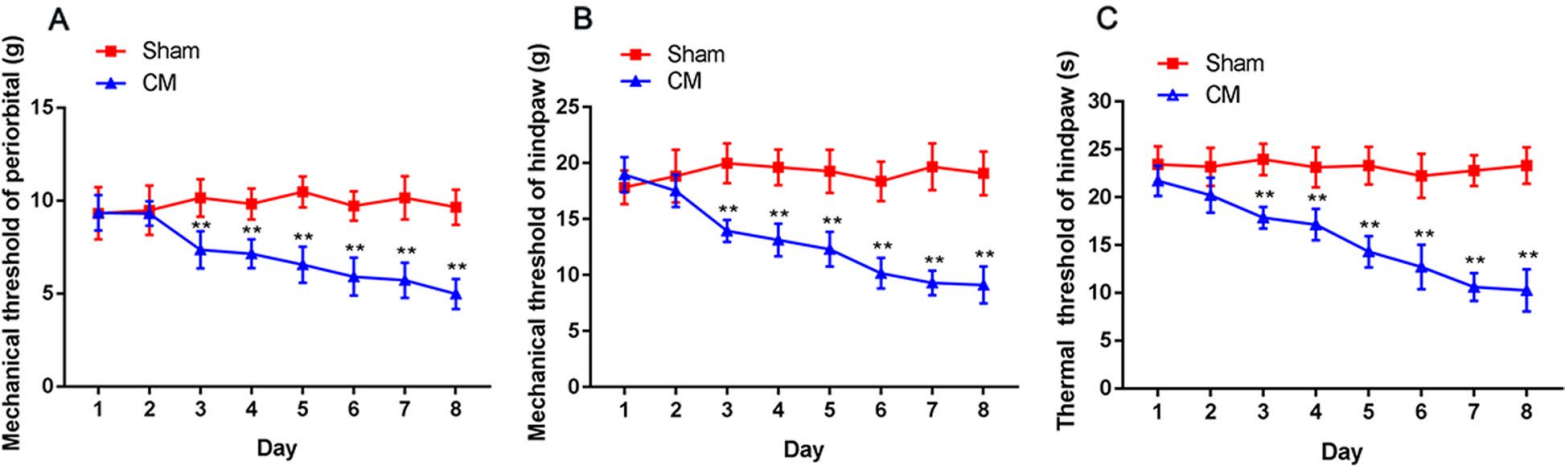
The influence of repeated dural IS stimulation on hyperalgesia in CM rats. **A** and **B** Compared with the Sham group treated with PBS infusion, the mechanical pain thresholds of the periorbital region and hindpaw in the CM group treated with IS infusion markedly reduced after the second day. (*n* = 10/group, **p < 0.01, vs Sham). **C** The thermal pain thresholds of the hindpaw in the CM group were visibly lower than those in the Sham group after the second day. (*n* = 10/group, **p < 0.01, vs Sham)

To evaluate hyperalgesia in CM rats to determine the reliability of the CM model, after dural infusion of PBS/IS, we determined the mechanical pain thresholds of the periorbital region, the mechanical pain thresholds of the hindpaw, and the thermal pain thresholds of the hindpaw. As shown in Fig. 2A-C, compared to the Sham group, a massive decline in the mechanical and thermal pain thresholds in the CM group appeared after the third day, and the mechanical and thermal pain thresholds gradually decreased with the increase in IS infusion, indicating that the development of hyperalgesia in CM was time dependent. The above results illustrate that we successfully established a reliable CM rat model.

### The change and distribution of cannabinoid receptors and HCN channels in the PAG of CM rats

**Fig. 3:**
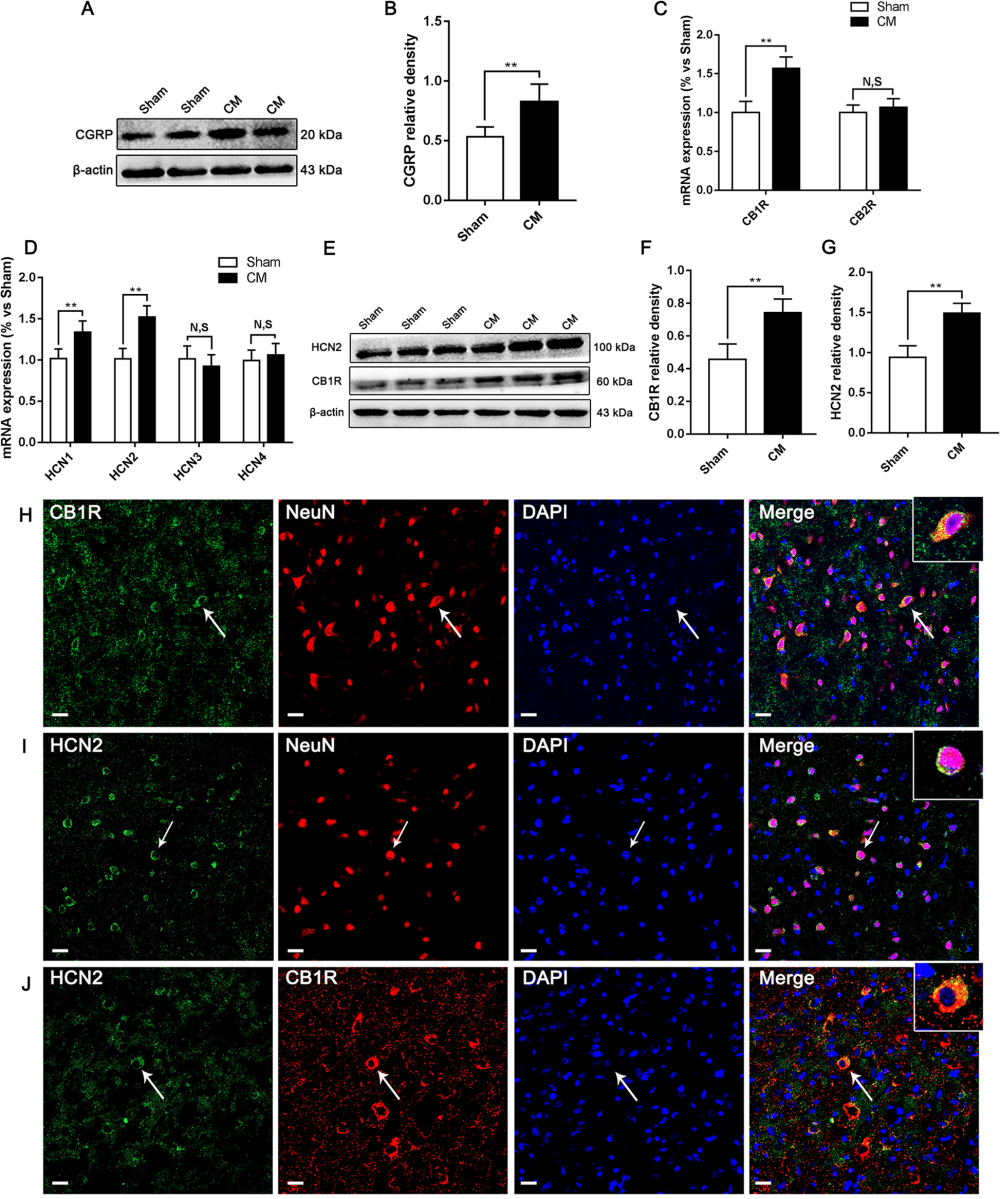
The change and distribution of cannabinoid receptors and HCN channels in the PAG of CM rats. **A** Representative western blots of CGRP. **B** The level of CGRP expression was substantially increased in the CM group compared to that in the Sham group. (*n* = 6/group, **p < 0.01). **C** The mRNA expression of CB1R in the CM group was markedly higher than that in the Sham group. The mRNA expression of CB2R was not markedly different between the CM and Sham groups; (*n* = 6/group, **p < 0.01, N, S, not significant). **D** The mRNA levels of HCN1 and HCN2 were substantially higher in the CM group than those in the Sham group. No change was found at the mRNA levels of HCN3 and HCN4 between the Sham and CM groups. (*n* = 6/group, **p < 0.01, N, S, not significant). **E** Representative western blots of CB1R and HCN2. **F-G** Compared with the Sham group, the protein levels of CB1R and HCN2 in the CM group were drastically increased. **H** Double immunofluorescence labeling of CB1R (green) and NeuN (red). In the PAG, almost all CB1R was expressed in neurons (as shown by the white arrow). (scale bar = 20 μm). **I** Double immunofluorescence labeling of HCN2 (green) and NeuN (red). HCN2 was found to be clearly expressed in neurons of the PAG (as shown by the white arrow). (scale bar = 20 μm). **J** Double immunofluorescence labeling of HCN2 (green) and CB1R (red) revealed that CB1R was coexpressed with HCN2 (as shown by the white arrow). (scale bar = 20 μm)

To explore the subtypes of cannabinoid receptors and HCN channels involved in the pathophysiological mechanism of CM, we used qRT–PCR to screen CB1R, CB2R, and HCN1-4 in the PAG. First, we validated the reliability of the model at the molecular level. As a classic biological marker in migraine [46], calcitonin gene-related peptide (CGRP) expression in the CM group was significantly higher than that in the Sham group **(**Fig. 3A and B**)**. Then, as shown in Fig. 3C and D, we found that the mRNA levels of CB1R, HCN1, and HCN2 in the CM group were materially increased compared to those in the Sham group, and there was no substantial difference in the expression levels of CB2R, HCN3, and HCN4 between the Sham and CM groups. To verify changes in CB1R and HCN2 expression in more detail, we employed western blotting to measure the protein levels of CB1R and HCN2. Consistently, the protein levels of CB1R and HCN2 were drastically elevated in the CM group compared to the Sham group (Fig. 3E-G). Furthermore, we used double immunofluorescence staining to explore CB1R and HCN2 localization in the PAG of Sham rats. The observation in Fig. 3H and I clearly shows that CB1R- and HCN2-immunopositive cells colocalized with NeuN (a marker of neurons) in the PAG. Simultaneously, we found that CB1R was coexpressed with HCN2 (Fig. 3J). Consequently, these results indicate that CB1R and HCN2 may be related to CM.

### The effect of NE and ZD 7288 on hyperalgesia in CM rats

**Fig. 4:**
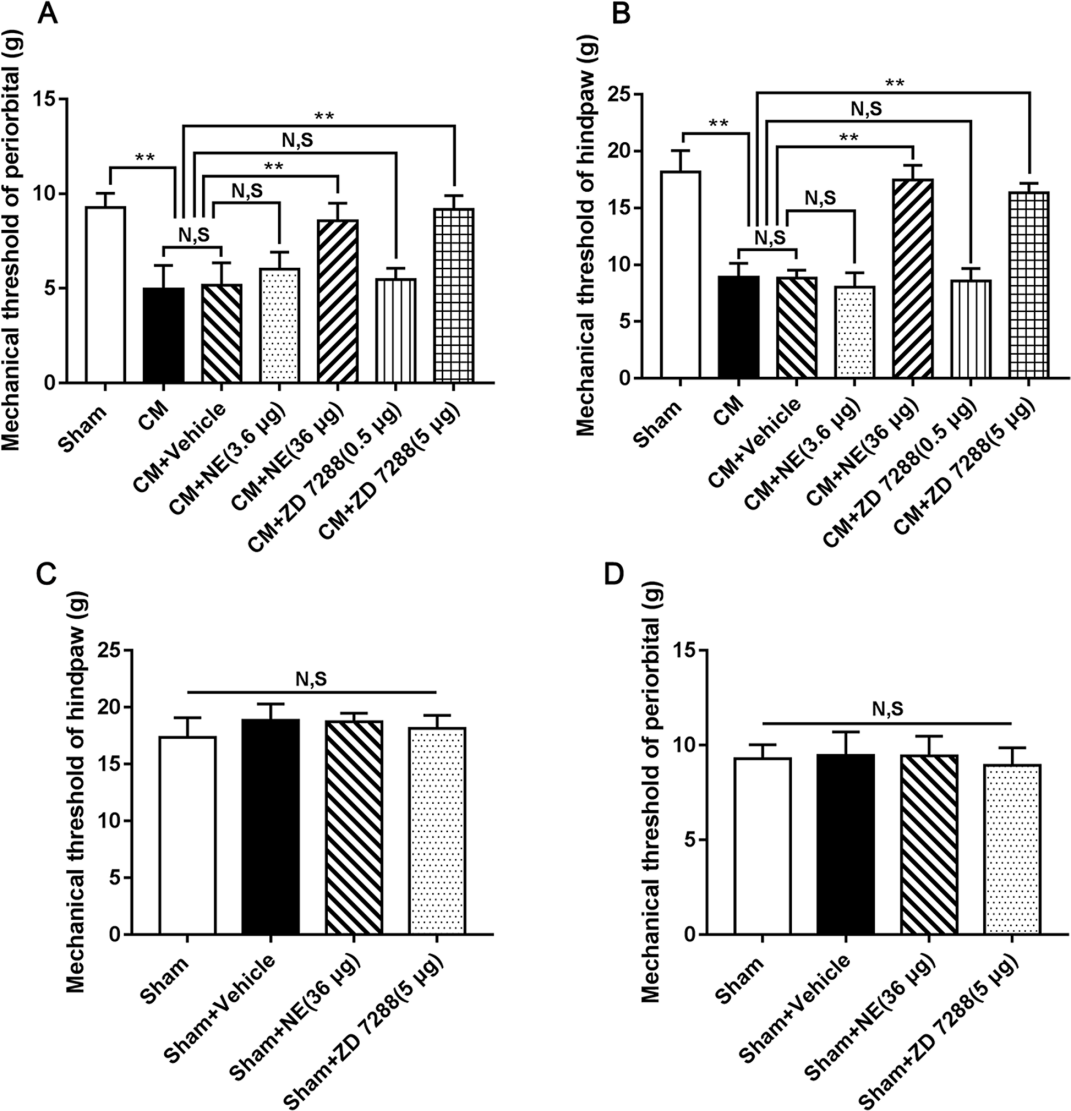
The effect of NE and ZD 7288 on hyperalgesia in CM rats. **A** and **B** The mechanical pain thresholds of the periorbital region and hindpaw in the CM group were substantially lower than those in the Sham group, and there was no obvious difference between the CM and CM + Vehicle groups. Compared to the CM + Vehicle group, the mechanical pain thresholds of the periorbital region and hindpaw in the CM + NE (36 μg) and CM + ZD 7288 (5 μg) groups were both profoundly elevated. The mechanical pain thresholds of the periorbital region and hindpaw did not show substantial differences among the CM + Vehicle, CM + NE (3.6 μg), and CM + ZD 7288 (0.5 μg) groups. (*n* = 6/group, **p < 0.01, N, S, not significant). **C** and **D**. No substantial difference was found in the mechanical pain thresholds of the periorbital region and hindpaw among the Sham, Sham + Vehicle, Sham + NE (36 μg), and Sham + ZD 7288 (5 μg) groups. (*n* = 6/group, N, S, not significant)

To further investigate the role of CB1R or HCN2 in hyperalgesia in CM rats, we tested the mechanical pain thresholds of the periorbital region and hindpaw in rats after low or high doses of the CB1R agonist NE or HCN2 blocker ZD 7288 were injected into the lateral ventricle. As shown in Fig. 4A and B, the mechanical pain thresholds of the periorbital region and hindpaw in the CM group were visibly lower than those in the Sham group. There was no substantial difference between the CM and CM + Vehicle groups. After separately injecting low or high doses of NE or ZD 7288, the high-dose NE (36 μg) and ZD 7288 (5 μg) markedly improved mechanical pain thresholds of the periorbital region and hindpaw of CM rats, reflecting that the application of high-dose NE (36 μg) and ZD 7288 (5 μg) relieved IS-evoked hyperalgesia in CM rats. However, the low-dose NE (3.6 μg) or ZD 7288 (0.5 μg) did not elevate the mechanical pain thresholds of the periorbital region and hindpaw. These results indicate that IS exposure triggered hyperalgesia in CM rats, but hyperalgesia could be remitted by activating CB1R or inhibiting HCN2. Accordingly, the high-dose NE (36 μg) and ZD 7288 (5 μg) were selected for subsequent studies.

To fully understand the effects of NE and ZD 7288 on the mechanical pain thresholds of the periorbital region and hindpaw, the NE or ZD 7288 was injected into the lateral ventricle of the Sham rats to eliminate their toxic effects. As shown in Fig. 4C and D, no significant difference was observed in the mechanical pain thresholds of the periorbital region and hindpaw in each group, suggesting that NE and ZD 7288 do not affect the periorbital and hindpaw mechanical pain thresholds in Sham group rats.

### The effect of NE on HCN2 and the pNR2B/CaMKII/pCREB pathway in CM rats

**Fig. 5:**
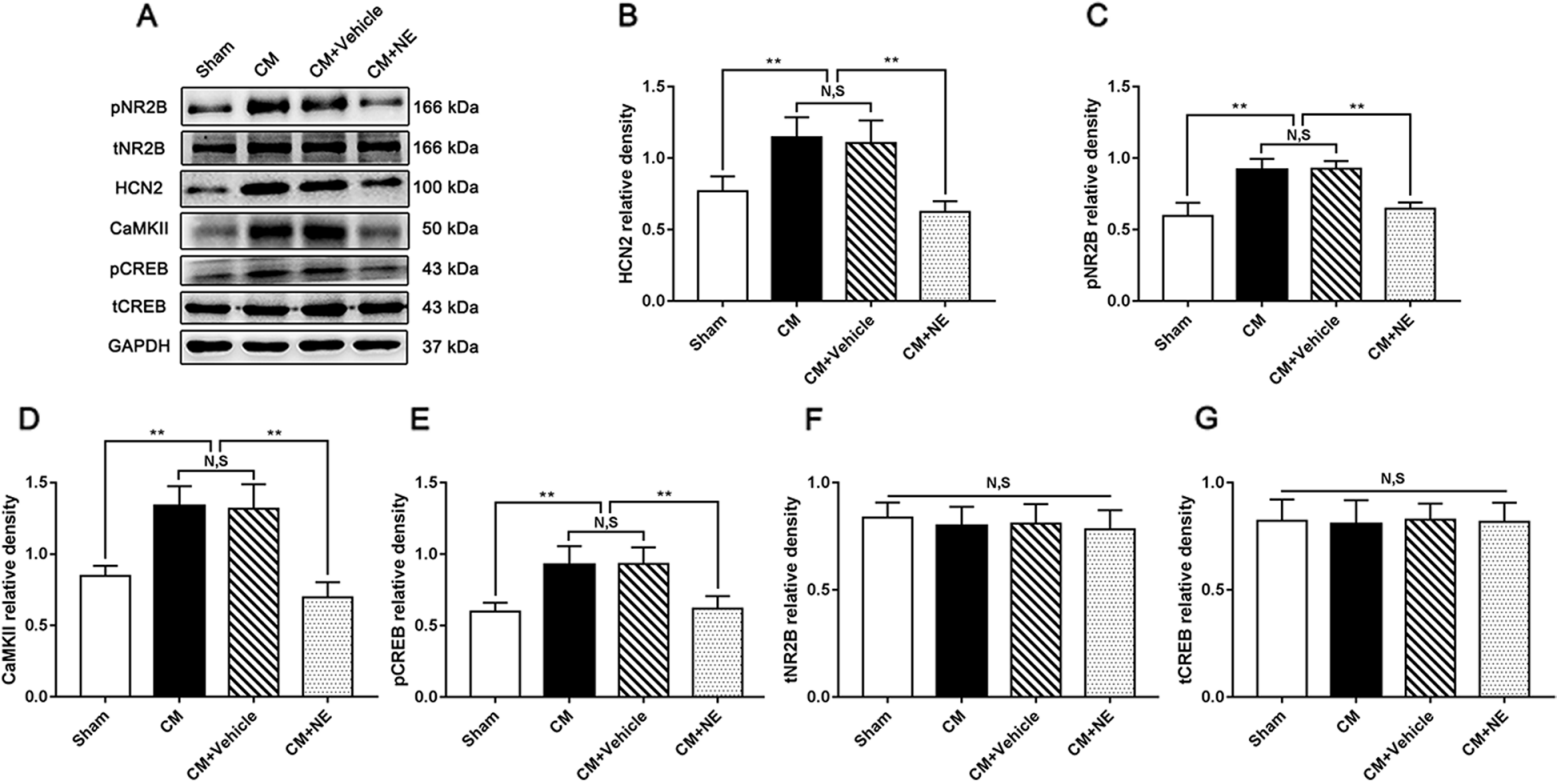
The effect of NE on HCN2 and the pNR2B/CaMKII/pCREB pathway in CM rats. **A** Representative western blots of HCN2, pNR2B, CaMKII, pCREB, tNR2B, and tCREB. **B-E** The expression levels of HCN2, pNR2B, CaMKII, and pCREB were obviously increased in the CM group compared with those in the Sham group, and there was no statistical difference between the CM and the CM + Vehicle groups. The HCN2, pNR2B, CaMKII and pCREB expression levels in the CM + NE group sharply reduced compared to the CM + Vehicle group. (*n* = 6/group, **p < 0.01, N, S, not significant). **F** and **G** There was no substantial difference in tNR2B and tCREB expression levels among the Sham, CM, CM + Vehicle, and CM + NE groups. (*n* = 6/group, N, S, not significant)

To investigate whether CB1R participates in CM by modulating HCN2 and the downstream pNR2B/CaMKII/pCREB pathway, we observed the expression of HCN2 and the pNR2B/CaMKII/pCREB pathway following the administration of NE. As shown in Fig. 5A-E, the expression levels of HCN2, pNR2B, CaMKII, and pCREB in the CM group were significantly higher than those in the Sham group. The expression levels of HCN2, pNR2B, CaMKII, and pCREB in the CM group were indistinguishable from those in the CM + Vehicle group. In the presence of NE, the expression levels of HCN2, pNR2B, CaMKII, and pCREB drastically reduced compared to those in the CM + Vehicle group. However, regardless of NE treatment, the total NR2B (tNR2B) and CREB (tCREB) levels remained unchanged in each group **(**Fig. 5A, F, and G**)**. Overall, the above results reveal that HCN2 and the downstream pNR2B/CaMKII/pCREB pathway were overactivated in the PAG of CM rats, activation of CB1R may prevent overactivation of HCN2 and the pNR2B/CaMKII/pCREB pathway in CM, and this process is independent of total NR2B and CREB.

### Regulation on central sensitization by NE in CM rats

**Fig. 6:**
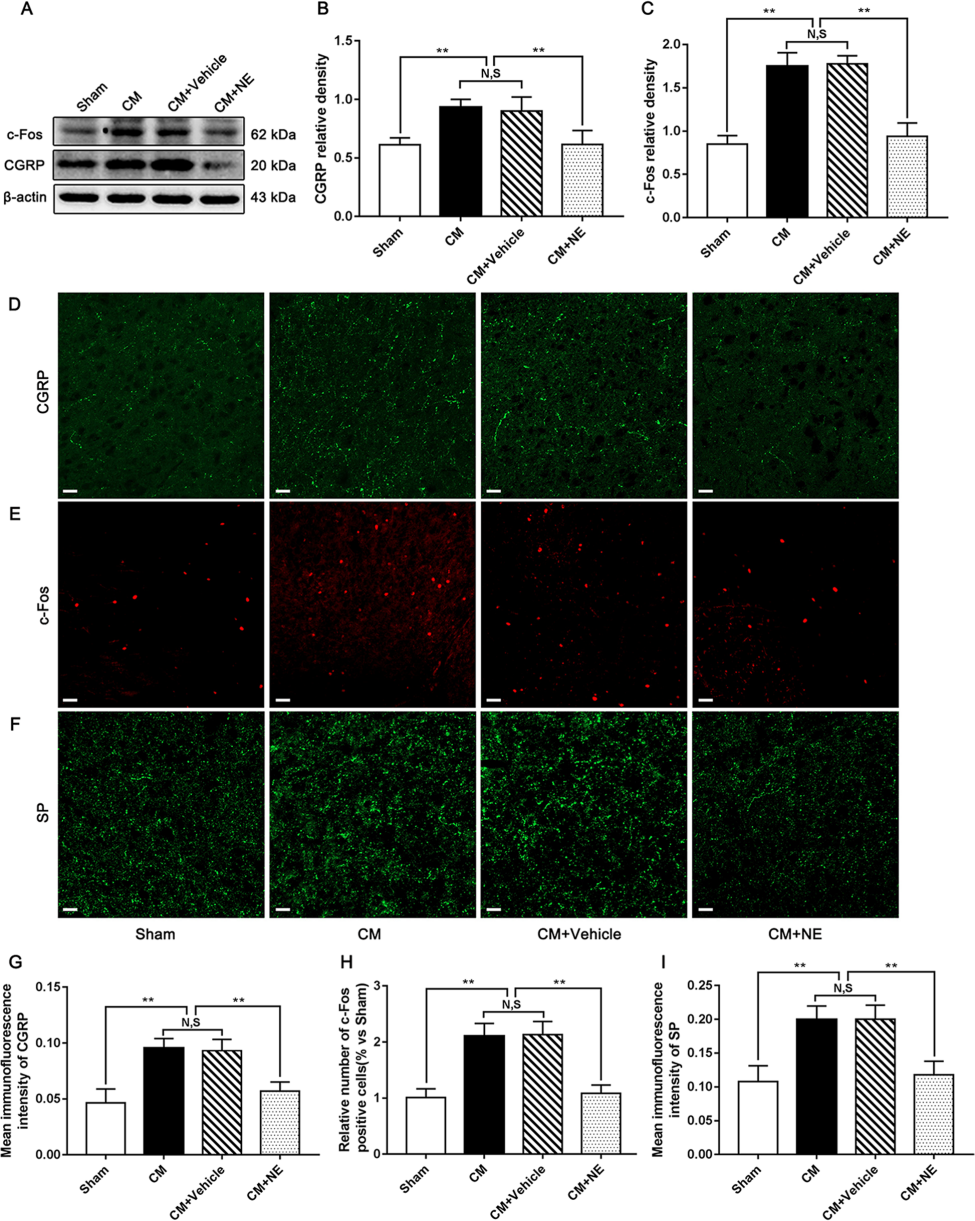
Regulation on central sensitization by NE in CM rats. **A** Representative western blots of CGRP and c-Fos. **B** and **C**. The protein levels of CGRP and c-Fos in the CM group were significantly higher than those in the Sham group, and there was no significant difference between the CM group and CM + Vehicle group. The CGRP and c-Fos expression levels in the CM + NE group were sharply reduced compared with those in the CM + Vehicle group. (*n* = 6/group, **p < 0.01, N, S, not significant). **D-F** Immunofluorescence staining of c-Fos, CGRP, and SP in the PAG. **G-I** The number of c-Fos-positive cells and the average fluorescence intensity of CGRP and SP were substantially higher in the CM group than those in the Sham group, and there was no statistical difference between the CM and CM + Vehicle groups. Compared to the CM + Vehicle group. The number of c-Fos-positive cells and the average fluorescence intensity of CGRP and SP were materially decreased in the CM + NE group. (*n* = 6/group, **p < 0.01, N,S, not significant, scale bar = 20 μm)

To clarify the adversarial mechanism of CB1R activation against central sensitization of CM, after the administration of NE, we measured the expression of central sensitization-associated indicators. Substance P (SP) and CGRP facilitate nociceptive transmission and participate in central sensitization by maintaining a hyperresponsive state. c-Fos is a classic marker of neuronal activation after noxious stimulation (20092559). The levels of CGRP, c-Fos, and SP were assessed by western blotting and immunofluorescence staining. As shown in Fig. 6A-C, the levels of CGRP and c-Fos were robustly increased in the CM group compared with those in the Sham group. No significant difference was observed between the CM and CM + Vehicle groups. Nevertheless, NE visibly reduced the increased levels of CGRP and c-Fos evoked by IS stimulation. Consistent with the western blotting results, we observed that IS infusion substantially elevated the number of c-Fos-positive cells and the average fluorescence intensity of CGRP and SP in CM rats, and there was no difference between the CM and CM + Vehicle groups. The number of c-Fos-positive cells and the average fluorescence intensity of CGRP and SP were sharply reduced under NE conditions (Fig. 6D-I**)**. Taken together, this expression pattern implies that activation of CB1R may alleviate central sensitization in CM.

### The effect of ZD 7288 on the pNR2B/CaMKII/pCREB pathway in CM rats

**Fig. 7:**
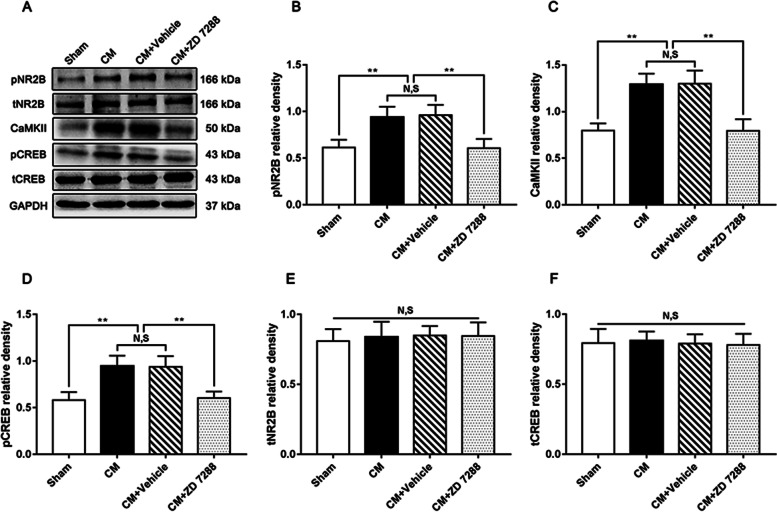
The effect of ZD 7288 on the pNR2B/CaMKII/pCREB pathway in CM rats. **A** Representative western blots of pNR2B, CaMKII, pCREB, tNR2B, and tCREB. **B-D** Compared with the Sham group, the levels of pNR2B, CaMKII, and pCREB in the CM group were profoundly elevated, and there was no change between CM and CM + Vehicle groups. Compared to the CM + Vehicle group, the levels of pNR2B, CaMKII, and pCREB in the CM + ZD 7288 group were visibly decreased. (*n* = 6/group, **p < 0.01, N, S, not significant). **E **and** F** There was no significant difference in tNR2B and tCREB expression among the Sham, CM, CM + Vehicle, and CM + ZD 7288 groups. (*n* = 6/group, N, S, not significant)

To clearly distinguish whether the link between CB1R and the pNR2B/CaMKII/pCREB pathway is related to HCN2 in CM, western blotting was employed to detect the expression of the pNR2B/CaMKII/pCREB pathway after ZD 7288 was injected intracerebroventricularly. As shown in Fig. 7A-D, the expression levels of pNR2B, CaMKII, and pCREB were markedly increased in the CM group, and no significant difference was observed between the CM and CM + Vehicle groups. Treatment with ZD 7288 substantially repressed the expression of pNR2B, CaMKII, and pCREB. Likewise, regardless of the application of ZD 7288, the tNR2B and tCREB levels remained unchanged in each group (Fig. 7A, E, and F). Overall, these results raise the possibility that the regulatory effect of CB1R on the pNR2B/CaMKII/pCREB pathway may be dependent on HCN2.

### Regulation of central sensitization by ZD 7288 in CM rats

**Fig. 8:**
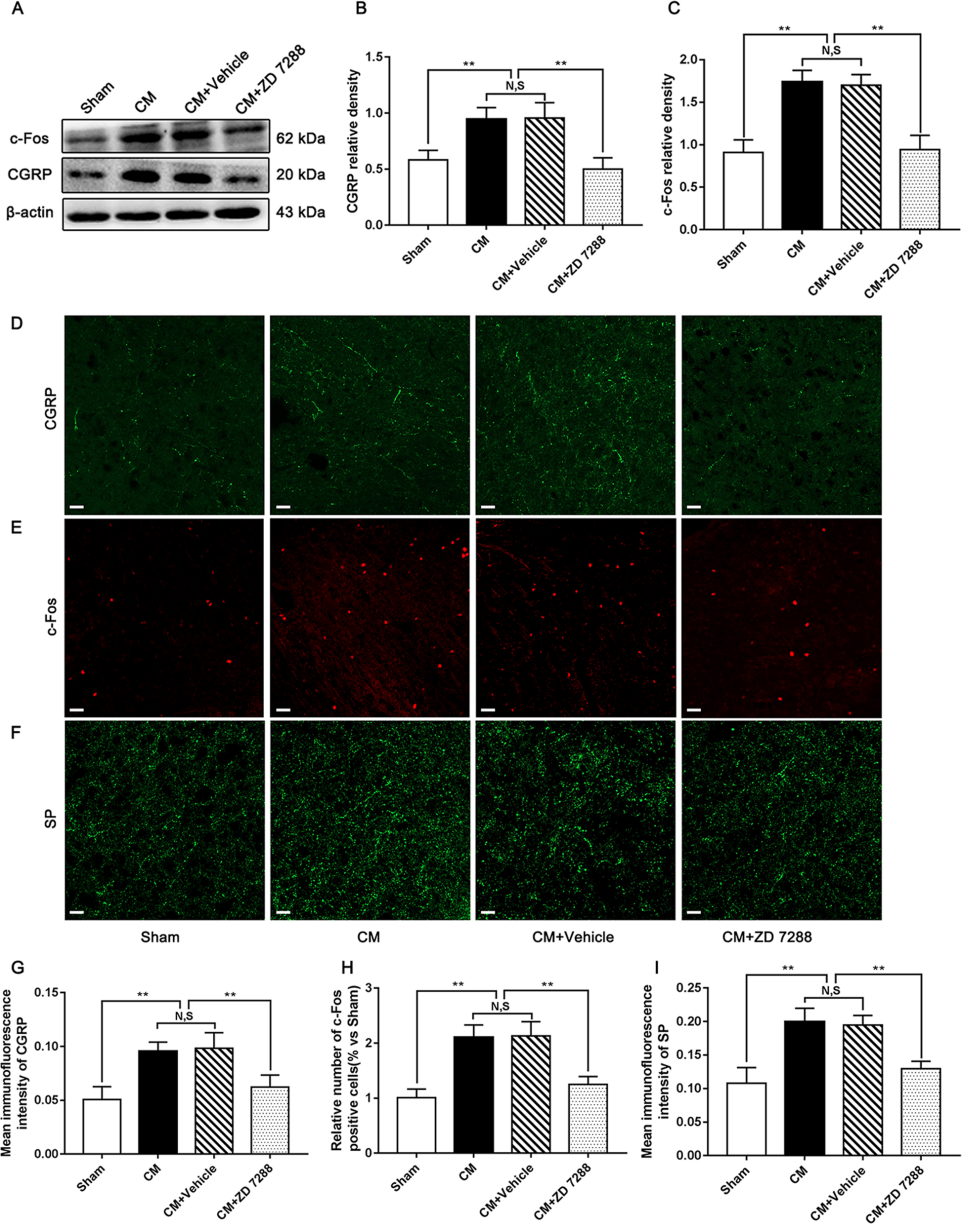
Regulation of central sensitization by ZD 7288 in CM rats. **A** Representative western blots of CGRP and c-Fos. **B** and **C** The protein levels of CGRP and c-Fos were significantly elevated in the CM group compared with those in the Sham group, and there was no statistical difference between the CM and CM + Vehicle groups. The expression levels of CGRP and c-Fos in the CM + ZD 7288 group drastically reduced compared to those in the CM + Vehicle group. (*n* = 6/group, **p < 0.01, N, S, not significant). **D-F** Immunofluorescence staining of c-Fos, CGRP, and SP in the PAG. **G-I** The number of c-Fos-positive cells and the average fluorescence intensity of CGRP and SP in the CM group were substantially increased compared to those in the Sham group, and there was no significant change between the CM and CM + Vehicle groups. The number of c-Fos-positive cells and the average fluorescence intensity of CGRP and SP in the CM + ZD 7288 group were visibly lower than those in the CM + Vehicle group. (*n* = 6/group, **p < 0.01, N,S, not significant, scale bar = 20 μm)

To assess the effect of HCN2 inhibition on central sensitization in the PAG of CM rats, after the administration of ZD 7288 intracerebroventricularly, western blotting and immunofluorescence staining were used to test the levels of CGRP, c-Fos, and SP. As shown in Fig. 8A-C, compared to the Sham group, the expression levels of CGRP and c-Fos were substantially higher in the CM group. Their levels in the CM group were indistinguishable from those in the CM + Vehicle group. The levels of CGRP and c-Fos expression in the CM + ZD 7288 group dramatically declined compared with those in the CM + Vehicle group. Similarly, the results obtained by immunofluorescence staining were fully consistent with those obtained by western blotting. In the presence of ZD 7288, the number of c-Fos-positive cells and the average fluorescence intensity of CGRP and SP were robustly reduced (Fig. 8D-I). In short, these observations consistently reflect that activation of CB1R may alleviate central sensitization by regulating HCN2 in CM.

### The influence of AM 251 and ZD 7288 on hyperalgesia in CM rats

**Fig. 9:**
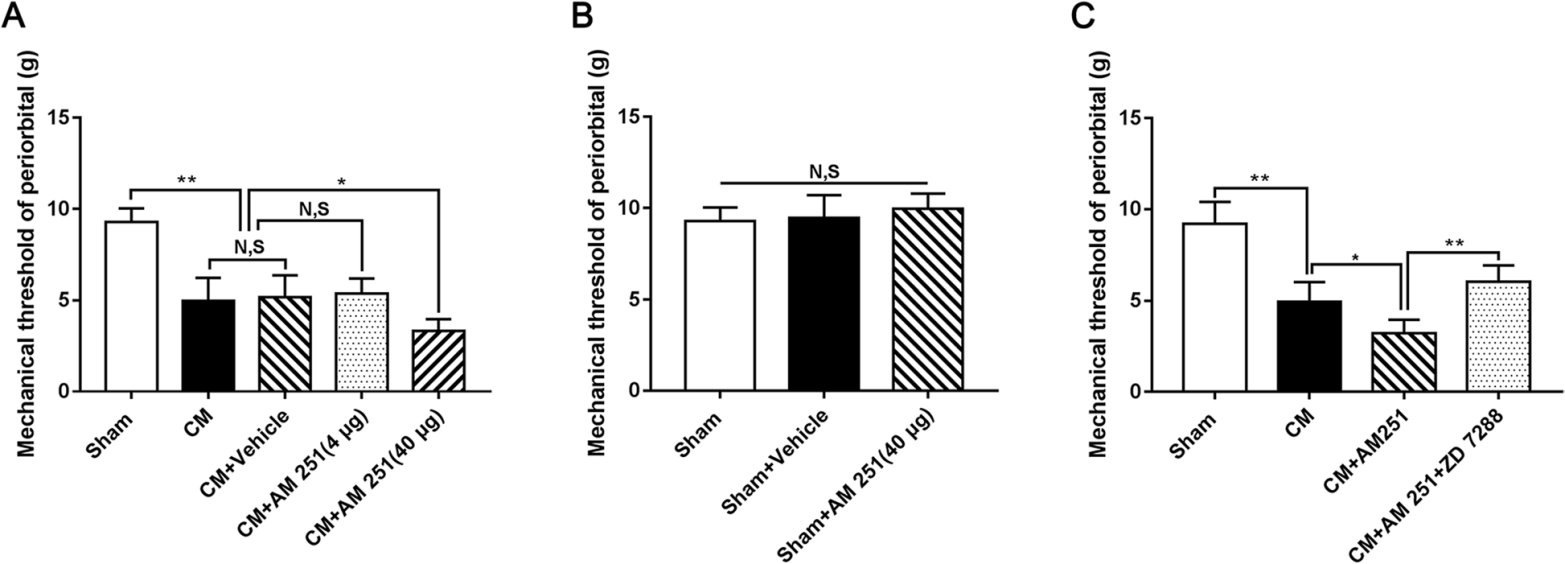
The influence of AM 251 and ZD 7288 on hyperalgesia in CM rats. **A** The mechanical pain thresholds of the periorbital region in the CM group were materially lower than in the Sham group, and there was no obvious difference between the CM and CM + Vehicle groups. Compared to the CM + Vehicle group, the mechanical pain thresholds of the periorbital region in the CM + AM 251 (40 μg) group were visibly decreased. The mechanical pain thresholds of the periorbital region in the CM + Vehicle group were indistinguishable from the CM + AM 251 (4 μg) group. (*n* = 6/group, *p < 0.05, **p < 0.01, N, S, not significant). **B** No substantial difference was observed in the mechanical pain thresholds of the periorbital region among the Sham, Sham + Vehicle, and Sham + AM 251 (40 μg) groups. (*n* = 6/group, N, S, not significant). **C** The mechanical pain thresholds of the periorbital region were robustly decreased in the CM group compared with those in the Sham group, and the mechanical pain thresholds of the periorbital region in the CM + AM 251 group were further drastically reduced compared to those the CM group. However, The mechanical pain thresholds of the periorbital region were significantly increased in the CM + AM 251 + ZD 7288 group compared to those in the CM + AM 251 group. (*n* = 6/group, *p < 0.05, **p < 0.01, N, S, not significant)

To clarify the role of HCN2 in CB1R-related hyperalgesia, ZD 7288 and AM 251 were simultaneously used by intraventricular injection after the effective dose of CB1R antagonist AM251 was screened by mechanical pain thresholds of the periorbital. Then, the mechanical pain thresholds of the periorbital were determined to evaluate hyperalgesia in CM rats. As shown in Fig. 9A, the mechanical pain thresholds of the periorbital region in the CM group substantially declined compared with those in the Sham group, and there was no change between the CM and CM + Vehicle groups. Treatment with high-dose AM 251 (40 μg) reduced the mechanical pain thresholds of the periorbital region, suggesting that the inhibition of CB1R further aggravated the degree of hyperalgesia in CM rats, while low-dose AM 251 (4 μg) was ineffective; thereby high-dose AM 251 (40 μg) was selected for subsequent studies. To eliminate the toxic effects of AM 251, AM 251 was applied in Sham rats, and the mechanical pain thresholds of the periorbital region remained unchanged under AM 251 conditions (Fig. 9B). Afterward, in the presence of both ZD 7288 and AM 251, we recorded the mechanical pain thresholds of the periorbital region. As shown in Fig. 9C, the application of AM 251 further reduced the mechanical pain thresholds of the periorbital region, but treatment with ZD 7288 abolished the AM 251-evoked decline in the mechanical pain thresholds of the periorbital region. Consequently, these data reflect that HCN2 is involved in the regulation of CB1R-related hyperalgesia in CM.

### The influence of AM 251 and ZD 7288 on the expression levels of pNR2B, CGRP, and c-Fos in CM rats

**Fig. 10:**
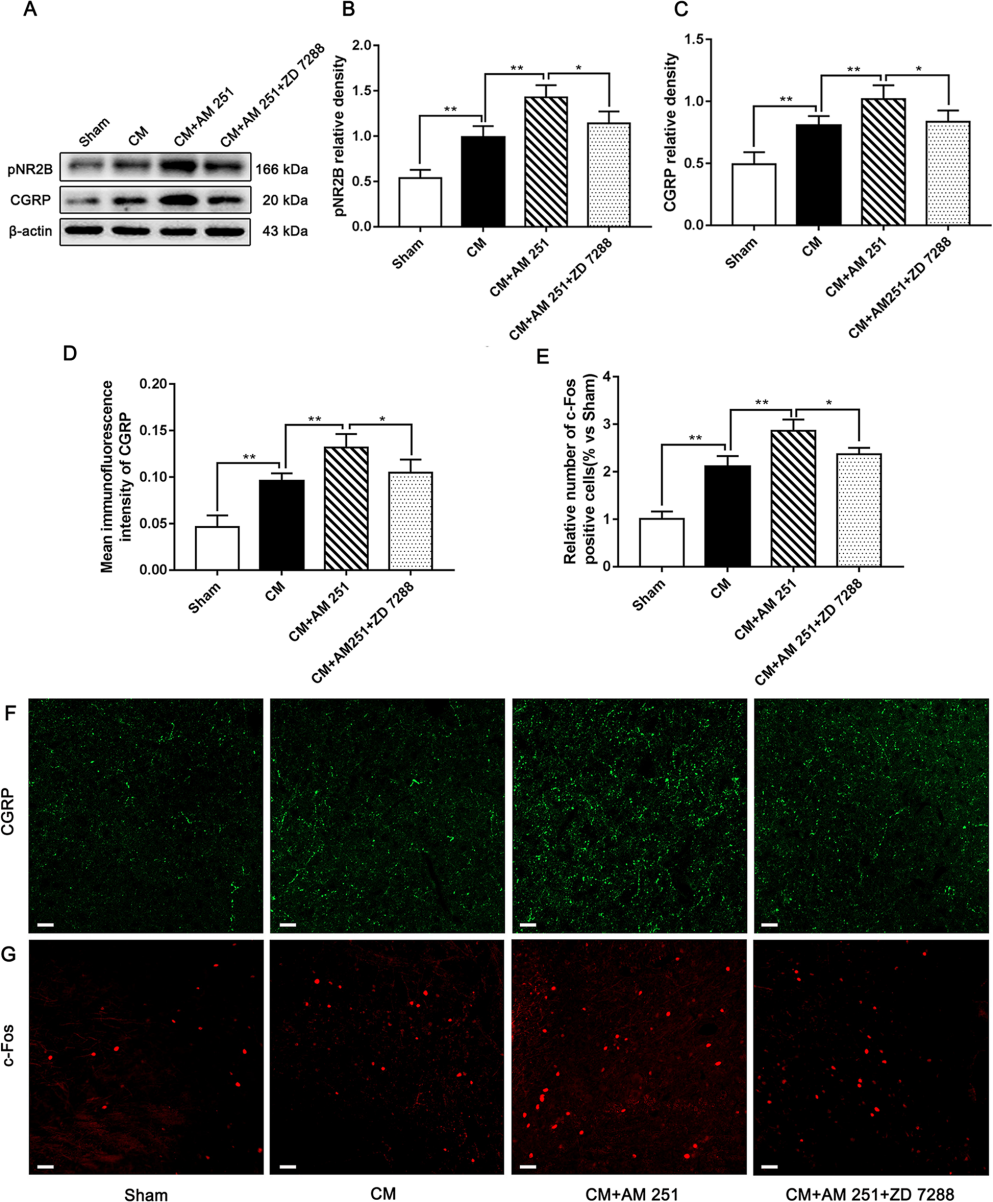
The influence of AM 251 and ZD 7288 on the expression levels of pNR2B, CGRP, and c-Fos in CM rats. **A** Representative western blots of pNR2B and CGRP. **B** and **C** The protein levels of pNR2B and CGRP were profoundly improved in the CM group compared with those in the Sham group, and there was no substantial difference between the CM and CM + Vehicle groups. Compared to the CM group, the levels of pNR2B and CGRP in the CM + AM 251 group were further increased. However, the expression of pNR2B and CGRP in the CM + AM 251 + ZD 7288 group was materially decreased compared to the CM + AM 251 group. (*n* = 6/group, *p < 0.05, **p < 0.01, N,S, not significant, scale bar = 20 μm). **D** and **E** The average fluorescence intensity of CGRP and the number of c-Fos-positive cells were statistically higher in the CM group than those in the Sham group. Compared with the CM group, the average fluorescence intensity of CGRP and the number of c-Fos-positive cells were visibly increased in the CM + AM 251 group. Importantly, the average fluorescence intensity of CGRP and the number of c-Fos-positive cells in the CM + AM 251 + ZD 7288 group sharply declined compared to the CM + AM 251 group. (*n* = 6/group, *p < 0.05, **p < 0.01, N,S, not significant, scale bar = 20 μm). **F-G** Immunofluorescence staining of CGRP and c-Fos in the PAG

To convincingly prove the vital role of HCN2 in the CB1R-regulated pNR2B/CaMKII/pCREB pathway and central sensitization, the levels of pNR2B, CGRP, and c-Fos were measured using western blotting and immunofluorescence staining. We observed that the levels of pNR2B, CGRP, and c-Fos in the CM group were materially higher than in the Sham group. Compared to the CM group, the levels of pNR2B, CGRP, and c-Fos were further increased in the CM + AM 251 group. Interestingly, when ZD 7288 and AM 251 were administered together, the application of ZD 7288 reversed the AM 251-induced elevation of pNR2B, CGRP, and c-Fos expression (Fig. 10A-C). The immunofluorescence staining results also confirmed this phenomenon. In the presence of AM251, the average fluorescence intensity of CGRP and the number of c-Fos-positive cells were substantially increased compared to those in the CM group. Similarly, the presence of ZD 7288 drastically alleviated the AM251-evoked elevation of the average fluorescence intensity of CGRP and the number of c-Fos-positive cells (Fig. 10D-G). Accordingly, these results further support the notion that HCN2 may be involved in the regulation of pNR2B and central sensitization by CB1R, and the underlying mechanism of the CB1R-mediated modulation of central sensitization was at least partially attributed to the contribution of the CB1R/HCN2/pNR2B pathway.

### Regulation of synaptic plasticity by NE or ZD 7288 in the PAG of CM rats

**Fig. 11:**
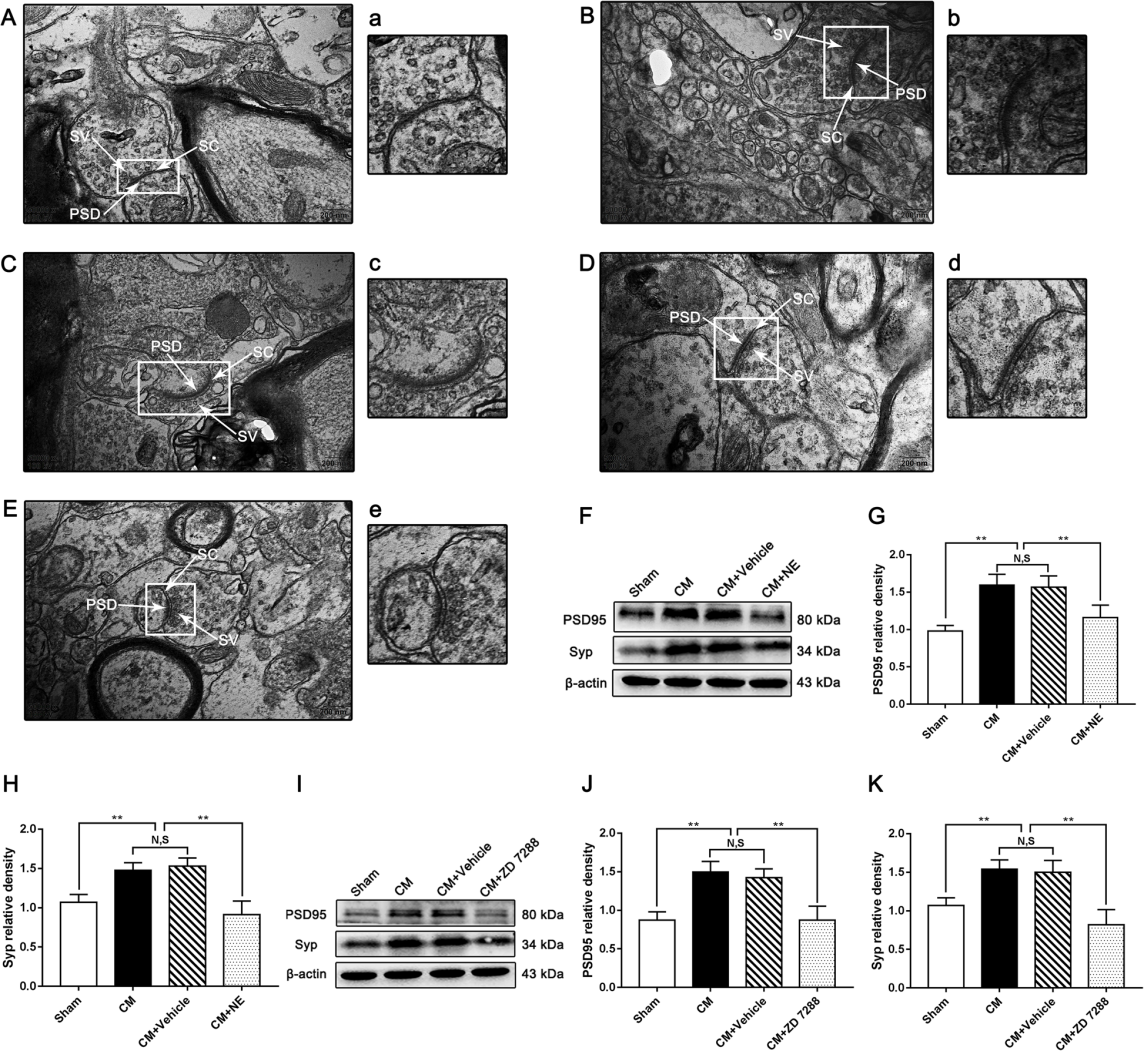
Regulation of synaptic plasticity by NE or ZD 7288 in the PAG of CM rats. **A-E** Synaptic ultrastructure in the four groups. A, a: Sham group; B, b: CM group; C, c: CM + Vehicle group; D, d: CM + NE group; E, e: CM + ZD 7288. PSD, postsynaptic density; SC, synaptic cleft; SV, synaptic vesicle. a-e show enlarged versions of the images in A-E. (Scale bars = 200 nm). **F** and **I** Representative western blots of PSD95 and Syp. **G** and **H** The protein levels of PSD95 and Syp in the CM group were drastically higher than those in the Sham group, and there was no significant difference between the CM group and CM + Vehicle groups. The PSD95 and Syp expression levels in the CM + NE group were seriously reduced compared with those in the CM + Vehicle group. (*n* = 6/group, **p < 0.01, N, S, not significant). **J** and **K** The protein levels of PSD95 and Syp were substantially higher in the CM group than those in the Sham group, and there was no statistical difference between the CM group and CM + Vehicle groups. Compared to the CM + Vehicle group, the levels of PSD95 and Syp were robustly decreased in the CM + ZD 7288 group. (*n* = 6/group, **p < 0.01, N, S, not significant)

The synaptic structure is the basis of synaptic plasticity. To investigate whether CB1R-HCN2 signaling can modulate synaptic plasticity in CM, we observed the synaptic ultrastructure of neurons in the PAG under TEM and measured the expression of synapse-associated proteins (postsynaptic density protein 95 (PSD95) and synaptophysin (Syp)) by western blotting after the administration of NE or ZD 7288. Representative images of the TEM results are shown in Fig. 11A-E. In the Sham group, the synaptic contour was clear, and abundant and transparent synaptic vesicles were observed in the anterior membrane region, while blurred synaptic clefts and presynaptic membranes were observed in the CM group. Additionally, the length of the active region, the curvature of the synaptic interface, the width of the synaptic cleft, and the thickness of the postsynaptic density were markedly increased in the CM group compared with the Sham group, reflecting higher synaptic transmission efficiency in the PAG of CM rats. No significant difference was observed between the CM and CM + Vehicle groups. The application of NE or ZD 7288 markedly reversed the IS-evoked changes in morphological indicators of the synapse (Table 3). In the western blotting results, the levels of PSD95 and Syp were significantly higher in the CM group than in the Sham group. There was no difference between CM and CM + Vehicle groups. Treatment with NE or ZD 7288 markedly reduced the IS-evoked elevation of PSD95 and Syp (Fig. 11F-K). Overall, these results suggest that CB1R-HCN2 signaling may be involved in the modulation of synaptic plasticity in central sensitization in CM.

**Table 3:**
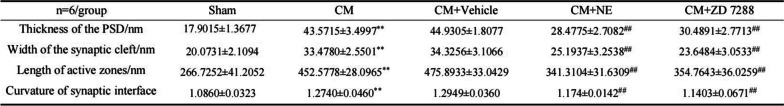
Synaptic ultrastructure parameters in the PAG neurons in each group

The thickness of the PSD, the width of the synaptic cleft, synaptic interface curvature, and active zones in the CM group significantly increased compared with those in the Sham group, while treatment with NE or ZD 7288 reversed the changes. There was no statistical change between the CM and CM + Vehicle groups. Data are presented as the mean ± SEM. (*n* = 6/group, **p < 0.01, vs Sham group; ##*p* < 0.01, vs CM + Vehicle group)

## Discussion

In this study, we used a rat model of CM by recurrent dural IS stimulation for seven consecutive days. The reliability of the model was proven by the upregulation of CGRP expression and the decreases in the mechanical and thermal pain thresholds after IS infusion, which is interpreted as a hyperalgesia phenomenon in CM. Based on the successful establishment of the model, we found increases in CB1R and HCN2 at the mRNA and protein levels in the PAG of CM rats, and CB1R and HCN2 were partially coexpressed on neurons of the PAG. Moreover, we also observed that pNR2B, CaMKII and pCREB expression dramatically increased in the PAG of CM rats, suggesting that the pNR2B/CaMKII/pCREB signaling pathway was activated after IS infusion. The increases in CGRP, c-Fos, and SP expression levels in the PAG of CM rats validated central sensitization. Furthermore, the activation of CB1R or suppression of HCN2 attenuated central sensitization triggered by IS stimulation through the pNR2B/CaMKII/pCREB pathway and ameliorated hyperalgesia in CM rats. Strikingly, the suppression of HCN2 prevented the further deterioration of central sensitization and hyperalgesia induced by the inhibition of CB1R by regulating pNR2B expression. Our research provides data regarding how the activation of CB1R participates in regulating central sensitization via HCN2 in CM rats and verifies the research hypothesis.

CB1R is observed on a wide range of neurons, including GABAergic, glutamatergic, cholinergic, etc. [47]. CB2R is primarily found in microglia or other cells of immune origin [48]. In pathological states, CB2R may also be expressed in neurons [49]. Endocannabinoids exert antinociceptive and antihyperalgesic effects by interacting with CB1R or CB2R. In most animal models of neuropathic and inflammatory pain, CB1R is observed to be increased in peripheral and central sensory pathways [50, 51]. In contrast, CB2 receptor expression is induced in the rat spinal cord in neuropathic but not inflammatory chronic pain models [52]. In addition, activation of CB1R rather than CB2R alleviates colonic nociception alone or in cooperation with a μ-opioid receptor [53]. As reported previously [54], the AM 251-induced blockage of CB1R markedly increases intradental capsaicin-triggered nociceptive response. CB1R seems to play a more vital role in the pain regulation mechanism than CB2R. Mounting evidence shows that the upregulation of CB1R enhances analgesic response to exogenous cannabinoids to contribute to the therapeutic effects of exogenous cannabinoids on pain [50]. This view is also confirmed in our study. CB1R mRNA and protein expression levels were both increased in CM rats, but CB2R expression was unchanged, suggesting that CB1R, rather than CB2R, may be involved in the development of CM. Moreover, the application of NE improved the pain thresholds of CM rats, while AM 251 further strengthened the IS-induced nociceptive response, indicating that CB1R can regulate IS-induced hyperalgesia in CM.

It is well known that activated CB1R represses the activity of adenylate cyclase (AC) by binding to the α-subunit of Gi/o protein [55], and then reduces the generation of intracellular cyclic adenosine monophosphate (cAMP) which promotes the opening of HCN channels by affecting the structure of the C-linker and cyclic nucleotide-binding domain of HCN channels [56]. Nevertheless, the sensitivity of HCN isoforms to cAMP varies greatly: HCN4 > HCN2 > HCN3 > HCN1 [57]. In our study, HCN1 and HCN2 expression at the mRNA level was upregulated in the PAG of CM rats, but the upregulation of HCN2 expression was more significant than that of HCN1. Moreover, considering that the response of HCN2 to cAMP is more sensitive than that of HCN1 to cAMP [58], we speculate that HCN2 is more likely to participate in the regulatory mechanism of CB1R to CM. Our results supported this speculation. We found that the protein expression of HCN2 robustly increased in the PAG of CM rats, and the activation of CB1R inhibited the IS-induced increase in HCN2, preliminarily indicating that CB1R has a regulatory effect on HCN2 and that this process may be dependent on cAMP. The activation of CB1R may inhibit the activity of HCN2 by reducing the cAMP level, thus suppressing the excitability of neurons and playing an analgesic role in CM.

Enhanced HCN2 expression has been previously found in many pain models [59]. HCN2 plays a crucial role in both inflammatory and neuropathic pain conditions by modulating the firing frequency of nociceptive sensory neurons [15]. A growing body of evidence shows the involvement of HCN2 in neuronal excitability, hyperalgesia, and synaptic plasticity in pain [60, 61]. Recently, a study reported that increased HCN2 expression is involved in oxaliplatin-evoked neuropathic pain by activating the NR2B-CaMKII-CREB cascade in spinal neurons, and oxaliplatin-induced LTP is suppressed by ZD 7288 in the spinal cord [19]. CaMKII, a serine/threonine protein kinase, can be activated by NMDA receptor-mediated Ca^2+^ influx [62]. Activated CaMKII phosphorylates CREB to participate in the regulation of synaptic plasticity [63]. Analogously, in our study, we observed that the pNR2B/CaMKII/pCREB pathway was overactivated in the PAG of CM rats, and the application of NE or ZD 7288 suppressed the activity of this pathway. Importantly, treatment with ZD 7288 abolished the AM 251-evoked elevation of pNR2B, indicating that CB1R may repress the pNR2B/CaMKII/pCREB pathway by regulating HCN2 signaling in CM. In the spinal dorsal horn, activation of the NMDA receptor stimulates Src family protein tyrosine kinase (SFK), which catalyzes NR2B at tyrosine residue 1472 specifically to increase the concentration of NR2B-containing NMDA receptor at synapses, thereby enhancing the phosphorylation of NR2B at tyrosine-1472 in homogenates of the spinal dorsal horn [64, 65]. This phenomenon was also observed in the PAG of CM rats, where pNR2B levels were increased, whereas the total NR2B content did not change. This may be related to SFK-mediated enrichment of NR2B at synapses. The accumulation of NR2B at synapses not only exaggerates NMDA receptor-mediated sensory transmission but also initiates a broad Ca^2+^-dependent signaling cascade in nociceptive neurons, resulting in enhanced neuronal excitability and response to noxious stimuli [64, 66].

Changes in synaptic plasticity were further explored by observing the synaptic microstructure and detecting the synaptic-associated proteins PSD95 and Syp. Receptor and synapse stabilization during development or after activity-driven synaptic potentiation both depend on PSD-95, which is critically involved in the modulation of LTP [67]. Syp is a type of calcium-binding glycoprotein that is widely distributed in the presynaptic vesicle membrane. Syp regulates the release of Ca^2+^-dependent neurotransmitters and is a response to the induction and dimension of LTP [68]. Our data show that the expression levels of PSD95 and Syp in the PAG of CM rats robustly increased. PSD95 and Syp expression sharply declined following activation of CB1R or suppression of HCN2, implying that the expression of synaptic function/structure-related proteins is closely correlated with CB1R and HCN2 in the PAG, CB1R-HCN2 signaling may have regulatory effects on synaptic plasticity via the pNR2B/CaMKII/pCREB pathway in CM. These views are further strongly supported by the TEM results. The thickness of the PSD, the length of the synaptic activity zone, the width of the synaptic cleft, and the curvature of the synaptic interface are four related indicators of the synaptic ultrastructure. Their elevations are the most intuitive demonstration of the increase in synaptic transmission efficiency in CM, and NE or ZD 7288 treatment reversed their elevations induced by IS infusion. These results further confirm that the activation of CB1R may modulate synaptic plasticity via HCN2-pNR2B signaling in CM.

The release of CGRP, an important neuropeptide consisting of 37 amino acids, leads to the development and maintenance of an overexcited state and thus facilitates nociceptive transmission, subsequently contributing to central sensitization, which constitutes the main pathogenesis of migraine [69]. The nuclear protein c-Fos is encoded by the immediate early gene c-fos and is rapidly expressed in response to different types of noxious stimulation in neurons. c-Fos directly promotes central sensitization through the transcriptional regulation of enkephalins [70]. SP participates in the modulation of nociceptive transmission and is vital in migraine pathophysiology, and the release of SP is proportional to the intensity and frequency of the nociceptive stimulus [71]. Intriguingly, phosphorylated CREB directly or indirectly increases the expression and release of the nociceptive neurotransmitters CGRP and SP by regulating transcription levels in the nucleus [72, 73], and the release of these nociceptive neurotransmitters activates neurons, thus increasing the expression of c-Fos [74]. In our study, we found visible elevations in the expression levels of CGRP, SP, and c-Fos in the PAG of CM rats, which is likely linked to the enhancement of processing of nociceptive information in CM. Activation of CB1R or inhibition of HCN2 markedly reversed the IS-evoked upregulation of CGRP, SP, and c-Fos. In particular, treatment with ZD 7288 abolished AM 251-evoked increases in CGRP and c-Fos levels. These data demonstrate that the activation of CB1R may reduce the expression of nociceptive transmitters and the intensity of neuronal excitation by inhibiting the HCN2-mediated pNR2B/CaMKII/pCREB pathway, in other words, alleviating central sensitization in CM.

Central sensitization manifests as pain hypersensitivity, especially hyperalgesia and allodynia [5]. The activation of nociceptive systems changes the threshold for nociceptive perception, producing an amplified response to noxious stimuli, and this is the pathological mechanism of hyperalgesia. Hyperalgesia is a characteristic component of neuropathic pain and is also observed in migraine patients [75, 76]. In CM, when sensitization spreads to the trigeminovascular neurons, the result is second-order neuron sensitization, which leads to scalp hypersensitivity or cutaneous allodynia. Generalized third-order neuron sensitization occurs when sensitization spreads to the thalamus, triggering extracephalic hypersensitivity (trunk and limbs) [77]. Most patients with migraine complain of cutaneous allodynia in the craniofacial region, often extending beyond the trigeminal receptive field to other parts of the body [78]. Punctate mechanical hyperalgesia and allodynia can be evaluated in practice by applying von Frey filaments with different forces, and thermal hyperalgesia in awake rats can be assessed by the Hargreaves test [79]. These two classical methods were used in our study to assess plantar and periorbital hyperalgesia in CM-associated central sensitization. In our research, both CB1 activation and HCN2 inhibition could effectively increase the pain thresholds of the rats, that is, both CB1 activation and HCN2 inhibition could alleviate hyperalgesia. Importantly, the application of AM 251 aggravated hyperalgesia in CM rats, while ZD 7288 reversed the effect of AM 251 on hyperalgesia in CM rats. These data further support the notion that activation of CB1R may alleviate central sensitization by inhibiting the HCN2-mediated pNR2B/CaMKII/pCREB pathway in CM.

## Conclusion

**Fig. 12:**
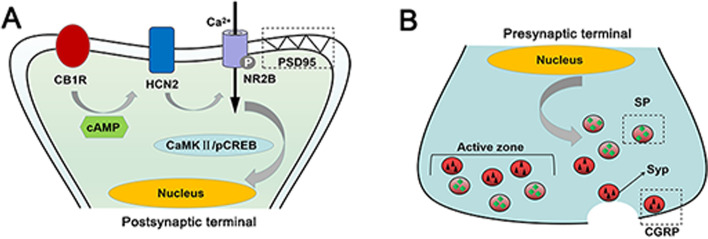
Schematic diagrams of CB1R-mediated regulation on central sensitization. **A** In CM, activation of CB1 inhibits HCN2 activity by reducing cAMP levels, thereby suppressing the pNR2B/CaMKII/pCREB pathway. **B** A decrease in intranuclear pCREB level alleviates central sensitization by regulating expression levels of synaptic proteins (PSD95 and SYP) and nociceptive transmitters (SP and CGRP) in CM

In conclusion, we explored the roles and relationship of CB1R and HCN2 in CM and paid close attention to the role of the HCN2-pNR2B signal in the CB1R-mediated regulatory mechanism of central sensitization in CM (Fig. 12), which helps to further identify CB1R as a promising candidate for future prevention of CM.

## Data Availability

Data can be made available upon request.

## Abbreviations

CM: Chronic migraine
IS: Inflammatory soup
PAG: Periaqueductal gray
CB1R: Cannabinoid type-1 receptor
CB2R: Cannabinoid type-2 receptor
HCN: Hyperpolarization-activated cyclic nucleotide-gated cation channel
NMDAR: N-methyl-D-aspartate receptor
NR2B: N-methyl-D-aspartate receptor subtype 2B
AC: Adenylate cyclase
cAMP: Cyclic adenosine monophosphate
CaMKII: Calcium-calmodulin-dependent kinase II
pCREB: Phosphorylation of cAMP-response element binding protein
CGRP: Calcitonin gene-related peptide
SP: Substance P
PSD95: Postsynaptic density protein 95
Syp: Synaptophysin
DAPI: 40,6-Diamidino-2-phenylindole
GAPDH: Glyceraldehyde-3-phosphate dehydrogenase
PBS: Phosphate-buffered saline
PVDF: Polyvinylidene difluoride
qRT–PCR: Quantitative real-time polymerase chain reaction
RIPA: Radioimmunoprecipitation assay
TNC: Trigeminal nucleus caudalis
TCC: Trigeminocervical complex
RVM: Rostral ventromedial medulla
SFK: Src family protein tyrosine kinase
WB: Western blotting
TEM: Transmission electron microscopy
